# Numerous Paragraphs of General Interest to the Profession

**Published:** 1893-08

**Authors:** 


### Medical News and Miscellany.

**Dr. I. N. Brewton,** of Floresville, as been appointed county physician of his (Wilson) county, Texas.

**Married.**—At Ben Wheeler, Texas, July 5, 1893, Dr. D. L. Saunders to Miss Alice Gray, daughter of Dr. A. J. Gray, of Ben Wheeler.

**Dr. J. D. Westervelt, jr.,** has removed from Corpus Christi to Alice, Texas, and engaged in general practice. We are pleased to learn that he is doing well.

**Professor William Goodell** has resigned the chair of gynecology at the University of Pennsylvania, and Dr. Charles B. Penrose has been elected to succeed him. Dr. Goodell becomes Professor Emeritus.

**A Thoroughly Competent Druggist,** twenty-eight years of age, married, desires a situation. Speaks Spanish and English, and has had ten years experience. Best testimonials as to character and habits and qualifications. Address "D," care this journal, Austin, Texas.

**The** following Southern medical schools graduated respectively this last spring: Medical Department of Tulane University, 93 men; Memphis Hospital Medical College, 90; Medical College of Alabama, 33; University of Tennessee, 14; University of Nashville and Vanderbilt University, 150; Medical College of Virginia, 25; Chattanooga Medical College, 30; University of Louisville, 189; University of Texas, 2.—*Ex.*

**The American Medical Editors** will have a meeting and banquet in Washington, on the evening of Monday, September 4th, the day preceding the assembling of the Pan-American Medical Congress. Dr. I. N. Love, of the *Medical Mirror*, St. Louis, is chairman of the Committee of Arrangements for Banquet. It is hoped that every medical editor will be present on the occasion. Address chairman of the Committee of Arrangements.

---

**Pharmacy** has been added to the curriculum of the Medical Department of the University of Texas by the Regents, and the appointment of a professor is now in order. We are not advised of the number of applicants for the chair,—presume there are several,—but the JOURNAL wants to suggest that Dr. James Kennedy, of San Antonio, could fill the position with distinguished ability, with honor to himself and credit to the State. We should like to see him appointed.

---

**Wanted:**—The address of the defunct concern recently known as the Robinson-Baker Advertising Bureau, lately doing business as alleged, at No. 107 Times building (or Pulitzer building) sixth floor, New York. Our draft for a small amount due for advertising a reputable firm which somehow came through this concern, came back unpaid, and endorsed "No such parties at address given." The firm wrote us that they paid Mr. R. B. Bureau the money for their ad. in this JOURNAL. Bill for sale at discount.

---

**The** legislature of Pennsylvania has passed an act establishing a medical council and three boards of medical examiners—a regular, a homeopathic, and an eclectic. Members of the boards are appointed by the Governor of the State, from lists furnished by each of the State Medical Associations, and each board will examine the candidates of its own school. After July 1, 1895, applicants must have studied medicine for at least four years. The different boards will be under the control of the medical council.—*Ex.*

---

**To the Physicians of Texas.**—Prof. Allen J. Smith, M. D., Professor of Pathology in the Medical Department of the University of Texas at Galveston, is investigating the nature and cause of *hemorrhagic malarial fever*, and is very desirous of extended opportunity to study it clinically. Prof. Smith desires the JOURNAL to say that he would be pleased to go any reasonrble distance from Galveston upon invitation of the attending physician, to examine any number of such cases, *provided* no *quinine* or other anti-malarial medication has as yet been employed in the case. This proviso is made to insure his seeing the true pathology of the disease, unmodified by drug action.—EDITOR.

**How** is this for an epitaph, which is said to be on a tombstone in a cemetery in Lincolnshire, England:

"Here lies the body of Johnny dear,
Snatched away by the diarrhœa."—*Ex.*

There is nothing whatever the matter with that epitaph; it places the blame of the "snatching" to the proper cause. But the following, taken from a tombstone in a burying-ground at Hoosic Falls, New York, is simply harrowing to a medical as well as to a poetic mind:

"Her body dissected by fiendish men,
Her bones anatomized;
Her soul we trust has risen to God,
Where no physicians rise."

---

**Surgical Instruments.**—A recent number of Meyer Bros. & Co.'s St. Louis *Drug Magazine* states that the Fort Worth Pharmacy Company, of Fort Worth, carries the largest stock of Surgical Instruments and mechanical curative devices in the Southwest. We are personally acquainted with these people and have a correct knowledge of their stock, fully endorse the above, and will further say, their prices we know to be as low as any of the Eastern houses, and that they are now giving good satisfaction to five hundred Texas doctors whom they number as their patrons. A year ago we asked the profession to aid us in building up this house—a home institution—where orders could be promptly filled and delivered. We have now to say that satisfactory results are being obtained. The Fort Worth Pharmacy Company are also agents for three manufactories of electric Batteries and two manufactories of Physicians' Chairs. Write to them for what you may want.

---

**The New Surgeon-General.**—The appointment by the President, of Dr. Geo. M. Sternberg to the position of Surgeon-General, U. S. A., meets with almost universal endorsement. It reflects credit on the President in that this is the very first instance, so far as we know, where the appointment has been made in recognition of any other claims than party fealty and party service. Dr. Sternberg labored for years in searching for the true cause of yellow fever and cholera, exposing his life in torrid climes; and in rendering this valuable service to the profession and the world he has justly earned unperishable fame. As said, he is the only man appointed Surgeon-General of the U. S. army of whom it can be said that he had distinguished himself in the cause of science. It looks a little unjust however to appoint any one to this position over the heads of those in line of promotion and who have served long years in expectation of it.

---

**Died.**—Mr. James A. Gibson, of Richmond, Fort Bend county, Texas, died on June 12, 1893, from tubercular meningitis. Mr. Gibson was a member of the first class to enter the full course in the medical department of the University of Texas at Galveston. He was a brilliant student, and highly appreciated by his fellow-students and teachers. The class of '93 passed the following resolutions of respect to his memory:

WHEREAS, It has seemed right—the wisdom of Providence, that our beloved and respected class-mate, James A. Gibson, should be called from our midst to the life which is hereafter and forever, be it

*Resolved*, That we thus publicly attest our appreciation of his firmness as a friend, his worth as a scholar, and his honor as a man; and that in respect to his memory there shall be draped for one month after the opening of the session a chair in each of the lecture rooms, and that the class shall for the same period wear crape in some suitable manner.

